# Study on the antiviral activity of San Huang Yi Gan Capsule against hepatitis B virus with seropharmacological method

**DOI:** 10.1186/1472-6882-13-239

**Published:** 2013-09-27

**Authors:** Haibo Xu, Qinghe Wu, Cheng Peng, Lijuan Zhou

**Affiliations:** 1 Department of Pharmacology, College of Pharmacy, Chengdu University of Traditional Chinese Medicine, 1166 Liutai Avenue, Wenjiang, Chengdu, Sichuan, P.R. China 611137; 2 Department of Pharmacology, College of Pharmacy, Guangzhou University of Chinese Medicine, Guangzhou, China; 3 Institute for Chinese Medicine Research, Sichuan Academy of Chinese Medicine Sciences, Chengdu, China

**Keywords:** Entecavir, Hepatitis B virus, San Huang Yi Gan capsule, Seropharmacology

## Abstract

**Background:**

Seropharmacology arising recently is a novel method of *in vitro* pharmacological study on Chinese herb using drug-containing animal serum. As seropharmacology possesses the advantages of experiments *in vitro* and *in vivo*, it is increasingly applied in pharmacological research on Chinese medicine. However, some issues of seropharmacology remain controversial and need to be clearly defined. San Huang Yi Gan Capsule (SHYGC) is a Chinese herbal formula with antiviral property against hepatitis B virus (HBV), but little is known about the mechanism underlying its anti-HBV activity. The aim of the present study was to elucidate the action mechanism of SHYGC using seropharmacological method and systematically address the methodology of preparing drug-containing serum.

**Methods:**

New Zealand rabbits were orally administrated SHYGC with various regimens, followed by preparation of SHYGC-containing rabbit sera with a variety of methods. After HBV-producing HepG2 2.2.15 cells were treated with SHYGC-containing sera or entecavir for 9 days, the levels of hepatitis B surface antigen (HBsAg) and HBV DNA and the activity of DNA Polymerase were determined in HepG2 2.2.15 cells-conditioned media.

**Results:**

An optimally standardized method of preparing drug-containing serum was raised for seropharmacology, with which SHYGC was demonstrated to suppress HBsAg expression, HBV DNA replication and DNA Polymerase activity in a dose-dependent fashion.

**Conclusions:**

This seropharmacological study shows SHYGC is a potentially powerful anti-HBV agent. Additionally, seropharmacology is a promising pharmacological method with a broad range of advantages, and it can be widely used in biomedical research, if combined with pharmacokinetics.

## Background

Seropharmacology is a novel method arising over the relatively recent years for pharmacological study on Chinese materia medica *in vitro* using drug-containing serum [1-5]. The essence of seropharmacology is administration of drug to experimental animal (rabbit, rat or mouse), followed by harvest of animal blood and conduction of *in vitro* pharmacological experiment with the drug-containing animal serum. This pharmacological method has the same advantages as the *in vitro* experiment, for instance, convenient control of experimental condition and exclusion of interference by internal factors, which facilitate in-depth study on the action mechanism of drug. More importantly, seropharmacology reflects the internal process (absorption, distribution, metabolism and excretion) of drug in the body, as the drug contained in the animal serum is in the form of bioactive metabolite with the truly pharmacological potency of interest after internal biotransformation. By building a bridge between the experiments *in vitro* and *in vivo*, seropharmacology is a perfect combination of the two experimental systems. As Chinese herb generally features numerous components, complex metabolism and multiple drug targets, it is an arduous task to clarify each ingredient. On the other hand, single component can not represent the whole herb in respect of efficacy. Therefore, seropharmacology is quite suitable to the study on Chinese medicine, and it is increasingly employed in pharmacological research on Chinese herb, especially Chinese herbal formula [2-5], However, seropharmacology maintains some elusive issues, particularly the method of preparing drug-containing serum, which need to be intensively elucidated.

Hepatitis B virus (HBV) is a major affliction which burdens people globally [6]. Currently, the approved therapies for HBV infection utilized in clinical setting consist mainly of alpha interferon [7] and nucleoside analogs, such as lamivudine [8] and entecavir (ETV) [9]. However, these antiviral agents often give rise to undesirable adverse reactions and their efficacies are severely compromised in most patients by development of antiviral resistance after prolonged therapy. Therefore, there is an urgent need for the development of new anti-HBV agents that are both safe and effective.

San Huang Yi Gan Capsule (SHYGC) is a traditional Chinese herbal formula composed of the water soluble extracts of 6 medicinal plants, including *Radix scutellariae*, *Rheum palmatum L.*, *Radix bupleuri*, *Radix astragali*, *Fructus schizandrae* and *Radix glycyrrhizae*. SHYGC shows remarkable potential of antiviral capability against HBV without appreciable side effects, and it significantly attenuates hepatitis B e antigen (HBeAg) level in the sera of patients with hepatitis B [2]. However, the underlying mechanism for anti-HBV activity of SHYGC has so far remained unknown.

Here, we systemically addressed the methodology of preparation of drug-containing serum and looked insight into the mechanism underlying the anti-HBV capacity of SHYGC using the seropharmacological method. Moreover, a standardized model is to hopefully set up for the seropharmacological study on traditional Chinese medicine.

## Methods

### Drugs and reagents

San Huang Yi Gan Capsule (SHYGC) containing 1.67 g of the water soluble extracts of 6 Chinese herbs, provided by Institute for Chinese Materia Medica, Guangzhou University of Chinese Medicine, was dissolved in double distilled water (ddH_2_O) at 500 mg/ml. The authors are prepared to supply SHYGC to anyone who wants to replicate the study. Entecavir (ETV), derived from Bristol-Myers Squibb Corporate (New York, NY), was dissolved in 100% dimethyl sulfoxide at 10 μM as a stock. Prior to experimental tests, ETV was diluted in cell culture medium, with working concentration of 10 nM. ^3^H-dTTP was supplied by PerkinElmer NEN Inc. (Massachusetts, USA). All other reagents were commercially available.

### Preparation of SHYGC-containing rabbit sera

All animal experiments were carried out in strict accordance with the China Experimental Animal Act 1988, and all animal research procedures were approved by the Research Ethics Committees of Guangzhou University of Chinese Medicine and Chengdu University of Traditional Chinese Medicine. New Zealand white rabbits weighing 2.0 ± 0.2 kg each were randomly arranged in 4 groups of 2 male and 2 female rabbits, and housed at one per cage in a ventilated, temperature- and humidity-controlled animal facility with 12 h light-12 h dark cycle, and fed with standard diet and distilled water *ad libitum*.

Rabbits of low, medium and high dose SHYGC groups were gavaged SHYGC in doses of 0.25 g/kg, 0.5 g/kg and 1.0 g/kg daily, while rabbits of vehicle group were gavaged the volume-matched ddH_2_O. Dose selection was based upon our previous study, in which SHYGC treatment significantly reduced HBeAg level in the sera of patients with hepatitis B [2]. SHYGC of daily dosage was divided into equal shares and given to rabbits once, twice or thrice per day for 7 days. On day 8, after 12 h of fasting, rabbits were subject to last administration. At 0.5 h, 1 h, 1.5 h, 2 h, 2.5 h, 3 h, 3.5 h and 4 h post-administration, blood were retrieved from rabbits, via ear artery or transcardial puncture, with anesthesia by injection of pentobarbital sodium at a dose of 40 mg/kg in the marginal ear vein. Then, sera were collected by centrifugation of blood at 2000 × g for 10 min at 4°C, and some of sera were heat-inactivated at 56°C for 30 min. Finally, all of rabbit sera were sterilized by filtration through 0.22 μm cellulose ester membranes.

### HepG2 2.2.15 cells culture and treatment

HepG2 2.2.15 cells were originally obtained from George Acs (Mount Sinai Medical Center, New York, N.Y.) and capable of constitutively producing and secreting HBV viral particles [10]. The cells were seeded on 24-well plates at 5 × 10^4^ cells per well and grown in 1 ml of minimal essential medium (MEM) supplemented with 10% fetal bovine serum, 380 μg of G418 per ml, 50U of penicillin per ml and 50 mg of streptomycin per ml in 5% CO_2_ atmosphere at 37°C. Forty eight hours post seeding, the cells approached 80% confluency and were treated for 9 days by 1 ml of MEM containing 2% fetal bovine serum, 380 μg of G418 per ml, 50U of penicillin per ml, 50 mg of streptomycin per ml, and 0.2 ml of SHYGC-containing rabbit sera, 0.2 ml of rabbit serum vehicle, or ETV at 10 nM. On day 10, the culture media conditioned by HepG2 2.2.15 cells were harvested and clarified by a 5-min centrifugation at 1000 × g at 4°C. The resultant supernatant media were processed for determination of hepatitis B surface antigen (HBsAg) expression, HBV DNA polymerase activity and HBV DNA level as below. Each test was performed in 8 wells in parallel.

### Enzyme-linked immunosorbent assay of HBsAg expression

Fifty microliters of above HepG2 2.2.15 cells-conditioned media were added on 96-well plates coated with anti-HBs (antibody to HBsAg), and underwent the measurement of HBsAg expression with enzyme-linked immunosorbent assay (ELISA) kits (Yueliangwan Biotechnology Co., Shenzhen, China), as per the manufacturer's specifications. HBsAg level was expressed as the value of sample (P) against negative control (N), represented by the ratio of sample OD_450nm_ (optical density at 450 nm) value to negative control OD_450nm_ value.

### Specific immunoprecipitation assay of HBV DNA polymerase activity

The HBV DNA polymerase (Pol) activity was sought in the above conditioned media by a minor modification of the specific immunoprecipitation method [11]. Briefly, 400 μl of samples were placed into tubes of test and blank control. After excessive anti-HBs were added, mixtures were incubated at 37°C for 60 min, followed by two washes with Tris–HCl buffer (0.2 M, pH 7.5). In test tubes were added 20 μl of 1 mg/ml pronase E, 20 μl of 0.2 M Tris–HCl buffer (pH 7.5) containing 1.5% NP-40 and 0.4% β-mercaptoethanol, and 20 μl of substrate containing 0.08 M MgCl_2_, 0.24 M NH_4_Cl, 1 mM of dATP, dCTP and dGTP each and 0.5 μCi ^3^H-dTTP. In blank control tubes, 20 μl of above substrate and 40 μl of 0.2 M Tris–HCl buffer (pH 7.5) were added. After incubation at 37°C for 4 h, the reaction mixtures were spun, and 50 μl of supernatants were spotted on glass fiber filters and assayed for trichloroacetic acid-precipitable radioactivity by scintillation counting. CPM (counts per min) values for test tubes were obtained by subtraction of those for blank control tubes.

### Dot blot hybridization assay of HBV DNA level

The detection of HBV DNA level in the above conditioned media was conducted with a non-radioactive dot blot hybridization assay (Xieheng Bioengineering Co., Shanghai, China), in light of the product handbook. Briefly, 50 μl of samples were applied to nitrocellu-lose membranes, followed by denaturation for 20 min. Then, the membranes were baked at 80°C for 1 h and prehybridized for 2 h at 42°C. After hybridization with digoxigenin-11-dUTP-labled probe DNA at 42°C for 24 h, the membranes were blocked by bovine serum albumin for 20 min at 42°C. The hybridized products were next detected by adding anti-digoxigenin-alkaline phosphatase antibody and the specified substrate. Blue-purple colors were precipitated in few minutes, and HBV DNA level was evaluated as percentage of the control.

### Statistical analysis

Data obtained in this study were processed to determine means and standard errors, and presented as mean ± SEM (standard error of the mean). The statistical significances between the mean values were assessed by one-way analysis of variance (ANOVA) or Student’s *t*-Test with SPSS software. A value of P *<* 0.05 was considered statistically significant.

## Results

### The methodology of preparing drug-containing serum

**Figure 1:**
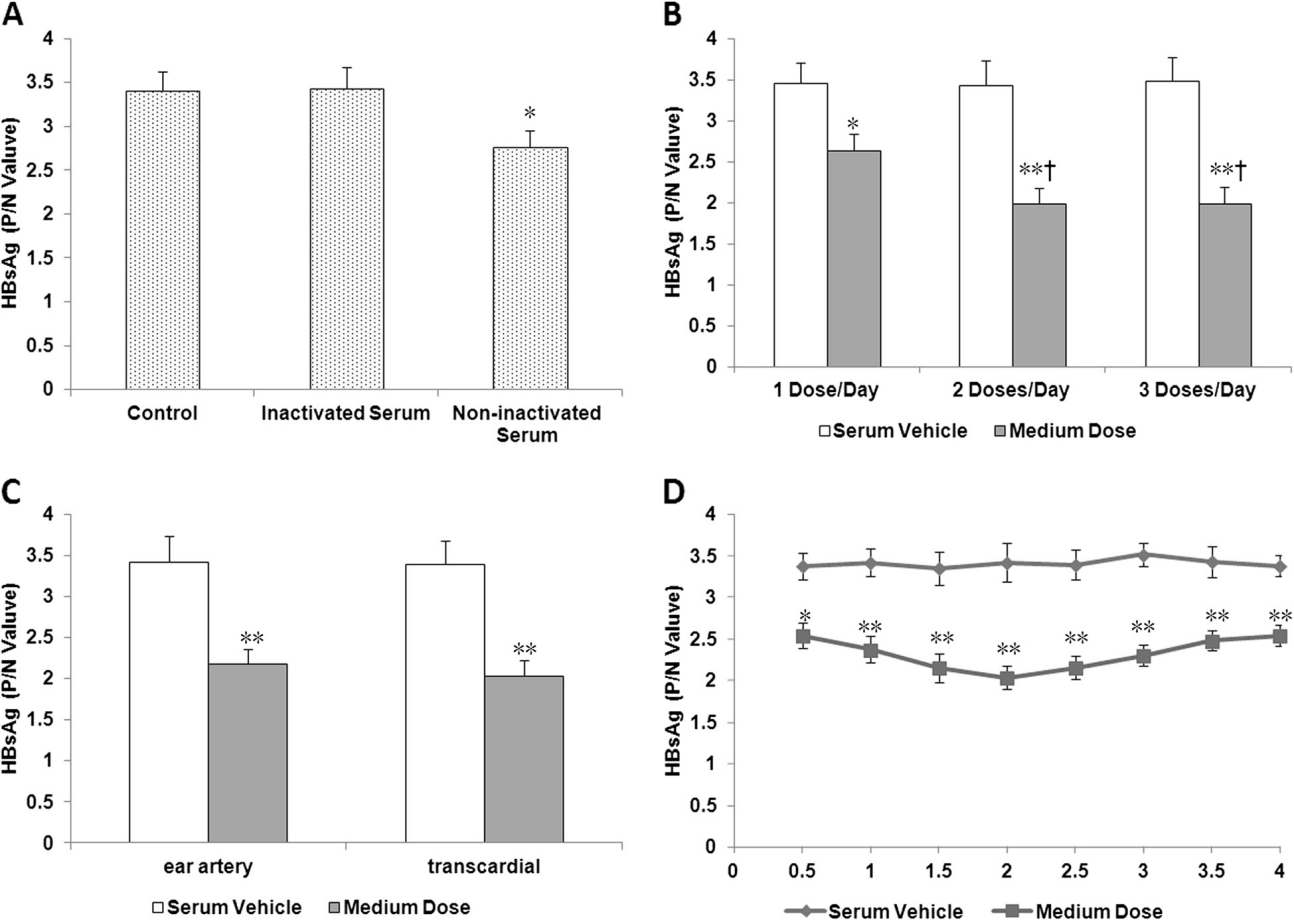
**Effects of SHYGC-containing rabbit sera, prepared with various methods, on HBsAg level in HepG2 2.2.15 cells-conditioned media.** Panel **A**, inactivated rabbit serum had no effect on HBsAg level in the conditioned media, while non-inactivated serum significantly mitigated HBsAg expression (* P < 0.05 *versus* Control and Inactivated Serum). Panel **B**, rabbit sera containing medium dose of SHYGC administrated with various regimens inhibited HBsAg expression (* P < 0.05 and ** P < 0.01 *versus* Serum Vehicle), and the potencies of SHYGC-containing sera prepared by administration twice daily and thrice daily were higher than once daily († P < 0.05). Panel **C**, medium dose SHYGC-containing rabbit sera harvested via ear artery and transcardial puncture both reduced HBsAg expression (** P < 0.01 *versus* Serum Vehicle). Panel **D**, medium dose SHYGC-containing sera collected at various time points after the final dose all attenuated HBsAg expression (* P < 0.05 and ** P < 0.01 *versus* Serum Vehicle).

We systematically studied the methodology of preparing drug-containing serum. First, the influence of serum itself on the result of pharmacological experiment was probed. We found that inactivated rabbit serum had no effect on HBsAg level in HepG2 2.2.15 cells-conditioned media, while non-inactivated serum significantly diminished HBsAg expression (Figure 1A). To preclude the effect of rabbit serum vehicle on HBV, all of rabbit sera containing or not containing SHYGC underwent inactivation at 56°C for 30 min, prior to the following experimental tests.

Given the same daily dosage, we wondered whether the administration regimen was a factor to affect the experimental result. Although all of medium dose SHYGC-containing rabbit sera reduced HBsAg expression, the potencies of drug-containing sera prepared by administration twice daily and thrice daily were higher than that of once per day. Additionally, no differences in the actions on HBsAg level were noted between twice daily and thrice daily (Figure 1B). In the following research, to facilitate the administration, rabbits received twice-a-day dosed treatment in the morning and at night to produce SHYGC-containing sera.

The approach of harvesting drug-containing sera from rabbits was then explored. Medium dose SHYGC-containing rabbit sera retrieved via ear artery and transcardial puncture both markedly lowered HBsAg expression, and drug-containing sera collected by these two methods revealed similar potency (Figure 1C). In the following experiments, for convenience of blood collection, drug-containing sera were harvested from rabbits via ear artery.

Finally, the optimal time point for collecting SHYGC-containing sera was determined. Medium dose SHYGC-containing sera harvested at all time points of 0.5 h to 4 h post-final administration displayed significant inhibition on HBsAg expression. While drug-containing sera collected at 2 h post-final administration exerted the strongest efficacy (Figure 1D). In the following assays, to obtain the highest potential of SHYGC, drug-containing sera were collected at 2 h post-final dose.

Consequently, an optimal and relatively standardized method was established to prepare drug-containing serum for seropharmacological research. In summary, drug of daily dosage was evenly divided into two portions and orally given to rabbits twice per day, in the morning and at night, for 7 consecutive days. On the 8th day, 2 h after the final administration, the rabbit sera was harvested via ear artery and subject to heat-inactivation at 56°C for 30 min. This comprehensive method of producing drug-containing serum was applied to all assays below.

### SHYGC-containing sera inhibits HBsAg expression

**Figure 2:**
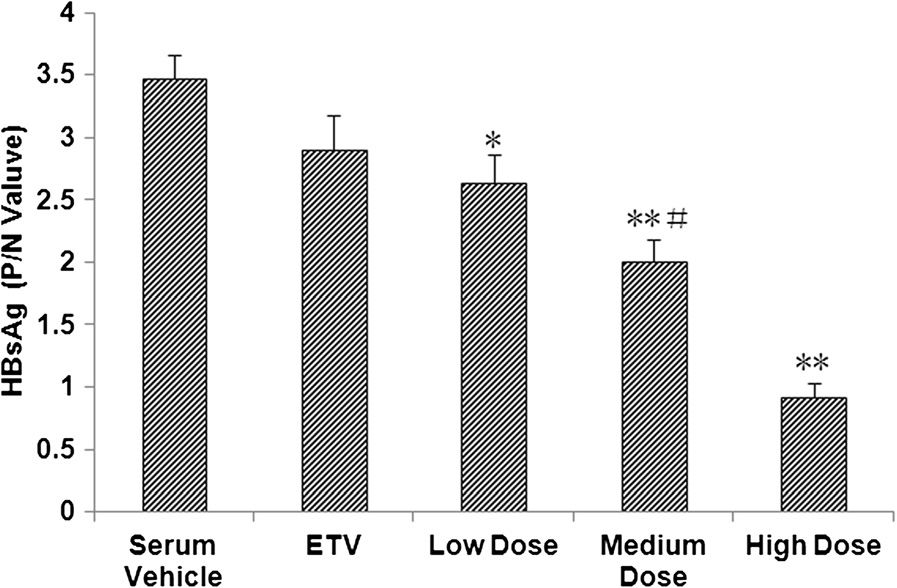
**Effects of SHYGC-containing rabbit sera, prepared with the optimal method, on HBsAg level in HepG2 2.2.15 cells-conditioned media.** Sera containing SHYGC at low, medium and high does all decreased HBsAg expression (* P < 0.05 and ** P < 0.01 *versus* Serum Vehicle), and the inhibitory potential of SHYGC showed dose dependent (# P < 0.05 *versus* Low Dose and High Dose).

Subsequently, we detected the effect of SHYGC on HBsAg level in HepG2 2.2.15 cells-conditioned media, using the above seropharmacological method. Compared to rabbit serum vehicle, rabbit sera containing SHYGC at low, medium and high does all attenuated HBsAg expression, and the inhibitory potential of SHYGC showed dose dependent. However, ETV did not significantly alter the HBsAg expression (Figure 2).

### SHYGC-containing sera inhibits HBV DNA Pol activity

**Figure 3:**
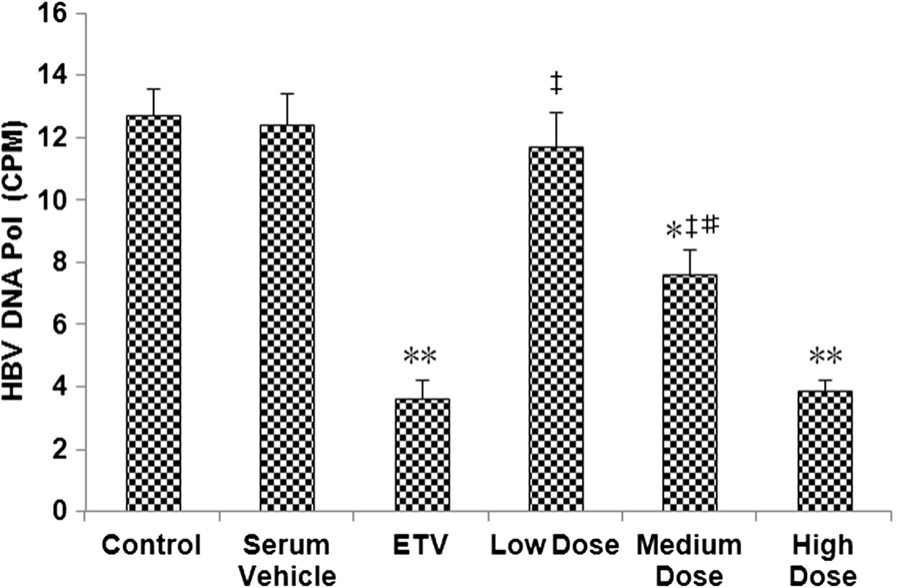
**Effects of SHYGC-containing rabbit sera, prepared with the optimal method, on HBV DNA Pol activity in HepG2 2.2.15 cells-conditioned media.** Sera containing medium and high doses of SHYGC and ETV all blunted HBV DNA Pol activity (* P < 0.05 and ** P < 0.01 *versus* Control and Serum Vehicle), and this kind of effectiveness of SHYGC was in a dose-dependent manner (# P < 0.05 *versus* Low Dose and High Dose). Treatment with high dose SHYGC-containing sera and ETV did not differ in the inhibition of DNA Pol activity (‡ P < 0.01 *versus* ETV).

In order to deeply elucidate the antiviral property of SHYGC against HBV, the action of SHYGC on HBV DNA Pol activity was assessed with a modified specific immunoprecipitation assay. Except low dose, rabbit sera containing medium and high doses of SHYGC and ETV all inhibited HBV DNA Pol activity, compared to the control and rabbit serum vehicle. The suppression of DNA Pol activity by SHYGC was in a dose-dependent manner. In addition, treatment with high dose SHYGC-containing sera and ETV did not significantly differ in the inhibition of DNA Pol activity (Figure 3).

### SHYGC-containing sera inhibits HBV DNA replication

**Figure 4:**
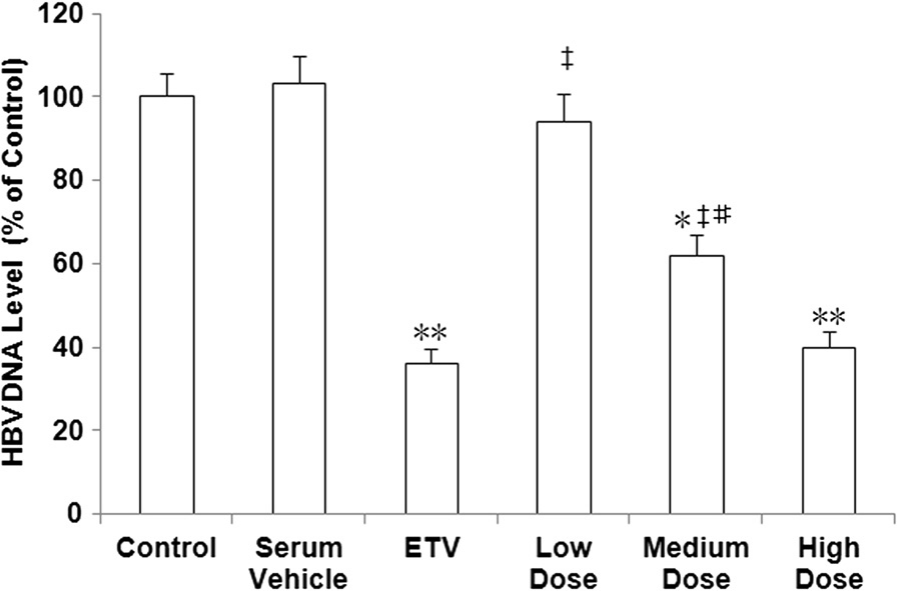
**Effects of SHYGC-containing rabbit sera, prepared with the optimal method, on HBV DNA level in HepG2 2.2.15 cells-conditioned media.** Sera containing medium and high doses of SHYGC and ETV alleviated HBV DNA replication (* P < 0.05 and ** P < 0.01 *versus* Control and Serum Vehicle), and this sort of efficacy of SHYGC was in a dose-dependent fashion (# P < 0.05 *versus* Low Dose and High Dose). High does SHYGC and ETV exhibited no differential potentials on the down regulation of DNA replication (‡ P < 0.01 *versus* ETV).

The appreciable effect of SHYGC on HBV DNA Pol activity drove us to further explore the potency of SHYGC on HBV DNA replication. We found that rabbit sera containing medium and high doses of SHYGC and ETV all lessened HBV DNA replication, compared to the control and rabbit serum vehicle. However, HBV DNA replication was not affected by low dose SHYGC-containing sera. The inhibition of DNA replication by SHYGC was in a dose-dependent pattern. Moreover, no difference in down regulation of DNA replication was noted between high does SHYGC and ETV (Figure 4).

## Discussion

One of the most annoying problems for pharmacological study is that the data of *in vitro* experiment sometimes differs greatly from that of *in vivo* test. The reasons for this problem may include the poor absorption of drug in the digestive track, biotransformation of drug to active metabolite only in the body, inactivation of compound during metabolism, and indirect action of drug through release of internal factors, such as endogenous hormone, cell factor, antibody, complement, and so forth. For Chinese herb, conventionally *in vitro* pharmacological method is addition of the soluble extract of medicinal plant in its original pharmaceutics to *in vitro* test system, such as cells, tissues, viruses, organs, etc. However, some flaws of this type of research method are always concerned. First, the pharmaceutical constituents (chemical, ion, pH value, impurity and osmotic pressure) are bound to alter the experimental condition. Second, in the original pharmaceutics may be not the truly active component after internal biotransformation and metabolism. Third, the pharmacological data from this *in vitro* experimental system should be validated as false positive and false negative results are likely displayed. Efforts have been made in recent years to resolve these above concerns with seropharmacology, which is to perform pharmacological experiment *in vitro* with drug-containing animal serum, which retains the real metabolite after physical biotransformation, and reflects the drug absorption into blood stream, internal metabolism and indirect action through endogenous factors. Seropharmacology also precludes the interference of pharmaceutical constituents on experimental outcome and promote the reliability of research data. Furthermore, seropharmacology is more benefiting to the study on Chinese herb, especially the Chinese herbal formula, that generally features the characters of complex components, multiple targets and various functions. Taken together, seropharmacology provides a proper strategy and a novel approach to pharmacological research of Chinese medicine, and possibly western medicine as well, by bridging the experiments between *in vitro* and *in vivo*.

The most important issue for seropharmacology is the preparation of drug-containing animal serum which has been highly controversial since the emergence of seropharmacology. Attempts were made in this report to set up an optimally standardized method of preparing drug-containing serum. According to this method, the selection of animal to treat with drug was on the basis of the amount of blood the animal can provide. Rather than rats and mice, rabbits that can offer more blood were recruited to produce SHYGC-containing sera. In addition, the twice-a-day and thrice-a-day dosed medications were more potent than once-a-day dosed medication, which may be due to single dose of large amount of drug exceeding the capability of absorption in gastrointestinal tract. With this method, serum drug concentration seemed to reach the steady state (Css) after 7 consecutive days of administration, because all of medium dose SHYGC-containing sera obtained at various time points significantly lessened HBsAg expression, although the exact concentration of the herbal formula in rabbit sera was not measured, mainly due to the technical problem.

Nowadays, the main anti-HBV agents approved by World Health Organization are nucleoside analogs and costly alpha interferon, which commonly show poor effects on drug-resistant mutated HBV and cause adverse reactions and rebound of HBV following the cessation of medication. Nucleoside analogs, such as lamivudine and ETV, exert the function of anti-HBV, chiefly by inhibition of HBV DNA replication, while no direct action is clearly confirmed on the expression of HBsAg and HBeAg, which are indicative of HBV infection and multiplication respectively [12]. Chinese herbs have been demonstrated recently to possess some advantages in the therapy of anti-HBV. For instance, HBeAg and HBV DNA are under negative seroconversions in chronic hepatitis B patients treated by *Radix astragali*, one of the herbs in SHYGC [13]. *Radix scutellariae*, another herb of SHYGC, suppresses both wild-type HBV and lamivudine-resistant mutated HBV gene expression and virus production in human hepatoma cells [14]. Based on our previous discovery that SHYGC is a potential anti-HBV agent by reducing HBeAg level in the sera of patients with hepatitis B, we showed that SHYGC dose-dependently diminished the expression of HBsAg, HBV DNA Pol activity and HBV DNA replication, which is in agreement with other reports on some herbs of SHYGC. For example, *Radix astragali* and *Fructus schisandrae* effectively blunt HBsAg expression in HepG2 2.2.15 cells and hepatocellular carcinoma cells respectively [15,16]. *Rheum palmatum L.* inhibits HBV DNA replication and DNA Pol activity in HepG2 2.2.15 cells [17,18]. Being a positive control, ETV, a guanosine nucleoside analogue with the activity against HBV reverse transcriptase, in this study drastically lowered HBV DNA replication and DNA Pol activity in HepG2 2.2.15 cells, consistent with previous reports [19-21], while no significant effect on HBsAg level was observed. In contrast, SHYGC presents a broad range of advantages by significantly suppressing the expressions of HBsAg and HBeAg, HBV DNA replication and DNA Pol activity, and it appears to be a promising candidate of HBV inhibitor in clinical practice. Concomitantly, seropharmacology was validated in our study to be applicable in pharmacological research of Chinese medicine.

Nevertheless, in this study inevitably remain some limitations, which should be under particular consideration. First, SHYGC concentration in rabbit sera was just assumed to attain the steady state (Css) at the time of collecting drug-containing sera, which was based on the principle of pharmacokinetics, in which serum drug concentration is gradually elevated to Css by multiple doses. Typically, if dosing interval is drug half-life (t_1/2_), the serum drug concentration will achieve Css in 5 to 7 t_1/2_ s. However, it is extremely difficult to obtain the exact t_1/2_ of SHYGC, that is composed of 6 Chinese herbs and a number of bioactive components with various t_1/2_ s, although the pharmacokinetics investigation of SHYGC would be supplementary to this study. On the other hand, Css is not essential for seropharmacological study, although the higher serum drug concentration is absolutely beneficial to the research. Second, in seropharmacological study including our research, healthy animals are commonly recruited to prepare drug-containing sera [1-5], but the animals with specific disease should be superior to healthy animals for this point, because the internal process of drug in animals with disease of interest is more close to that in human patients, and animals of disease model could differ significantly from healthy animals in the biotransformation and metabolism of drug, which may be a critical factor to influence the ingredients of drug-containing serum. However, there is a long way to go to establish various animal models of specific disease for seropharmacological research. Third, notwithstanding seropharmacology is a combination of *in vivo* and *in vitro* tests, it can not entirely represent the study *in vivo*, because the true effectiveness of drug in the body may be affected by other factors, such as the level of drug receptor in the body, the affinity of drug to receptor, the physical response to drug, etc.

## Conclusions

Taken together, the antiviral property of SHYGC against HBV is strongly underpinned, which merits further investigation of the action mechanism and rigorously designed clinical trial in future. In this study, a relatively standardized model for seropharmacological research is established, and seropharmacology is shown to be a promising method for pharmacological study on drugs, in particular Chinese medicine. Combining with pharmacokinetics, seropharmacology can be broadly applied in biomedical research with bright prospect.

## Abbreviations

ANOVA: One-way analysis of variance; Css: Steady state concentration; ETV: Entecavir; HBeAg: Hepatitis B e antigen; HBsAg: Hepatitis B surface antigen; HBV: Hepatitis B virus; Pol: Polymerase; SEM: Standard error of the mean; SHYGC: San Huang Yi Gan Capsule.

## Competing interests

The authors declare that they have no competing interests.

## Authors’ contributions

HBX, QHW and CP conceived the study. HBX and LJZ carried out the experiments. HBX, QHW, CP and LJZ analyzed the data. All authors contributed to the manuscript preparation and approved the final manuscript.

## Pre-publication history

The pre-publication history for this paper can be accessed here:

http://www.biomedcentral.com/1472-6882/13/239/prepub
